# Stage-Dependent Changes in Subchondral Trabecular Bone Mechano-Structure in Primary Knee Osteoarthritis with Varus Malalignment

**DOI:** 10.3390/jfmk11020210

**Published:** 2026-05-26

**Authors:** Andreja Baljozovic, Uros Andjelic, Marko Vujacic, Marko Dimitrijevic, Danijela Djonic, Zoran Bascarevic, Jelena Jadzic

**Affiliations:** 1 Institute of Orthopedics Banjica, Faculty of Medicine, University of Belgrade, Mihaila Avramovica 28, 11000 Belgrade, Serbia; a.baljozovic@gmail.com (A.B.); vujacic13marko@gmail.com (M.V.); markodimi@gmail.com (M.D.); zoran.bascarevic@iohbb.edu.rs (Z.B.); 2 Center of Bone Biology, Faculty of Medicine, University of Belgrade, Dr. Subotica 4/2, 11000 Belgrade, Serbia; urosandjelic1@gmail.com (U.A.); danijela.djonic@med.bg.ac.rs (D.D.)

**Keywords:** knee osteoarthritis, KL grade, micro-CT, micro-indentation, total knee arthroplasty

## Abstract

**Background:** Reports on subchondral bone mechano-structure in individuals with various stages of knee osteoarthritis (KOA) are limited and often conflicting in contemporary literature. Our study aimed to assess differences in subchondral trabecular bone mechano-structure across late KOA stages in a homogenous group of patients with varus malalignment (confirmed by negative hip-knee-ankle-angle values). **Methods:** This retrospective cross-sectional study included micro-computed tomography scanning and Vickers micro-hardness testing of 90 bone samples (30 femoral and 60 tibial) collected from 15 adult patients with primary KOA undergoing total knee arthroplasty (TKA). The Kellgren–Lawrence grading system was used to assess the severity of KOA lesions in the included individuals, and bone samples were divided into the following groups: moderate KOA (42 samples from seven patients, age: 70 ± 7 years, females: 3/7) and end-stage KOA (48 samples from eight patients, age: 70 ± 6 years, females: 5/8). **Results:** Our data revealed site-specific sclerotic alterations in subchondral trabecular bone mechano-structure (thicker trabeculae, coupled with higher bone mineral content and increased bone micro-hardness) in individuals with end-stage KOA compared to moderate KOA, supporting its role in KOA pathogenesis beyond the exclusive cartilage degeneration effect. Our data also revealed that most heterogeneous subchondral trabecular mechano-structure was present in bone samples obtained from the medial part of the tibial and femoral condyle, revealing the substantial effect of mechanical loading during varus knee malalignment. **Conclusions:** Observed site-specific alterations in subchondral bone mechano-structure in individuals with end-stage KOA supported the role of subchondral sclerosis in primary KOA pathogenesis beyond its exclusive effect on cartilage degeneration.

## 1. Introduction

Knee osteoarthritis (KOA) is a progressive disease characterized by degeneration of articular cartilage and subchondral bone, leading to chronic pain and impaired knee joint function, which predominantly affects the elderly population [1]. Epidemiological studies indicate a continuous increase in its prevalence, primarily due to population aging and increased life expectancy. In 2021, the estimated global KOA prevalence was approximately 374 million cases, with projections suggesting it may nearly double to 650 million by 2045 [2]. Data from the United States suggest that the lifetime risk of developing radiographic and symptomatic KOA by the age of 85 has been estimated at up to 45%, with a higher risk observed in females, obese individuals, and patients with a history of knee injury [3].

Imaging analysis of KOA-induced osteochondral changes remains an active area of contemporary research. Various techniques are currently available to assess osteochondral changes in KOA, each with its own advantages and limitations. Some clinical methods, such as dual-energy X-ray absorptiometry (DXA) and quantitative computed tomography (QCT), primarily assess bone mass, providing areal bone mineral density (aBMD) and volumetric bone mineral density (vBMD), respectively [4,5]. Since two individuals can have the same bone mass at a specific skeletal site but greatly different bone strength, it is important to examine how other hierarchical bone structural features contribute to bone biomechanics [6,7]. Magnetic resonance imaging has shown limited precision for detailed bone microarchitectural assessment [8,9], while high-resolution peripheral quantitative computed tomography (HR-pQCT) is constrained by availability, high cost, and scanner limitations, particularly in patients with obesity, shorter lower limbs, or restricted knee motion [10,11]. Micro-computed tomography (micro-CT) allows for detailed 3D analysis of bone microarchitecture at high spatial resolution (with a limited field of view) in human samples from patients who have undergone knee replacement surgery [12].

Both experimental and in vivo studies have provided evidence of a strong biochemical and biomechanical interdependence between articular cartilage and subchondral bone [13]. Increasing evidence suggests that microstructural changes in the subchondral bone directly influence the load distribution experienced by the overlying cartilage, while it has been known that structural alterations in knee joint cartilage may consequently influence the mechanical strength of underlying subchondral bone [14,15]. Biomechanically, early subchondral bone microstructural alterations may reduce shock-absorbing capacity and, therefore, predispose the knee joint to accelerated cartilage loss [15]. Altered load transfer on cartilage and subchondral bone accelerates KOA progression, as the homeostasis and integrity of articular cartilage rely on its biochemical and biomechanical interactions with the underlying bone [13]. Microstructural analyses of human specimens with late-stage KOA have shown increased subchondral bone volume and trabecular thickening, accompanied by articular cartilage degeneration [16,17,18]. Still, it is important to note that most previous studies have focused on assessing only tibial subchondral bone microarchitecture, whereas alterations in femoral subchondral bone across different KOA stages are scarce. Furthermore, contemporary research on the impact of osteoarthritis on the mechanical properties of subchondral bone is limited and often contradictory [14]. Namely, pioneering studies on hip osteoarthritis revealed increased mechanical properties of subchondral bone [19], whereas later studies indicated reduced mechanical properties in these patients [19,20]. On the other side, increased mechanical properties of tibial subchondral bone (in terms of increased shear modulus and elastic modulus) were recently noted in patients with KOA [14,21]. It has also been highlighted that knee malalignment plays a key role in load-distribution-induced microstructural changes in subchondral trabecular bone, representing a significant factor in KOA progression [4,12,22]. Since bone mechanical properties are heterogeneous (in terms of plastic and elastic deformation) and highly site-dependent [23,24], implying their substantial contribution to postoperative implant stability [25], further research is required to fully elucidate subchondral bone mechano-structural alterations across different KOA stages, including their potential relationship with knee malalignment. Based on the previously stated, we hypothesized that the knee joint subchondral bone mechano-structure would differ across different KOA stages in a homogeneous group of patients with varus knee malalignment. Thus, our study aimed to assess potential differences in subchondral bone mechano-structure of the distal femur and proximal tibia between different stages of primary KOA (Kellgren–Lawrence stage 3 and Kellgren–Lawrence stage 4) in patients with varus knee malalignment who underwent TKA.

## 2. Materials and Methods

### 2.1. Study Design and Bone Sampling Procedure

This retrospective cross-sectional study assessed 90 formaldehyde-fixed bone samples (30 femoral and 60 tibial) collected from 15 adult patients with primary KOA who underwent TKA at the Institute of Orthopedics Banjica, between 2023 and 2025. Participants were excluded from this study based on the following criteria [12]:

1. Traumatic injury to the knee joint;
2. Ligament instability;
3. Receipt of intra-articular treatments such as viscosupplementation or corticosteroid knee injections;
4. Chronic bone-affecting comorbidities, including chronic liver or renal disease, parathyroid, adrenal, gonadal dysfunction, active solitary or metastatic cancer, and osteomyelitis;
5. Chronic alcohol or substance abuse;
6. Permanent immobility or being bedridden;
7. Previous fragility fractures;
8. Use of anti-resorptive medications, glucocorticoids, hormonal replacement, and chemotherapy;
9. Inability to provide informed consent or cognitive impairment.

**Figure 1:**
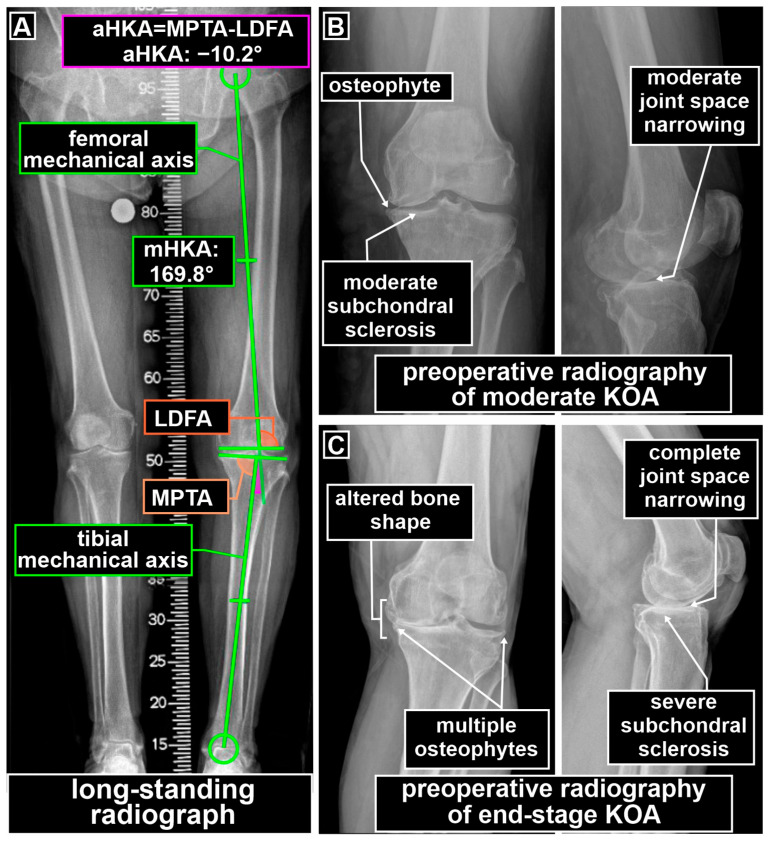
Representative preoperative radiographies of included individuals with primary KOA undergoing TKA. Readers should note that varus malalignment in the included individuals was confirmed by preoperative lower limb deformity analysis using long-standing radiographs (**A**). Patient with moderate KOA (Kellgren–Lawrence stage 3) preoperatively exhibits moderate osteophytes, joint space narrowing, and subchondral sclerosis (**B**), while a patient with end-stage KOA (Kellgren–Lawrence stage 4) preoperatively exhibits large osteophytes, severe to complete joint space narrowing, severe subchondral sclerosis, and a visibly altered shape of the femur and tibia (**C**). Abbreviations: KOA—knee osteoarthritis; TKA—total knee arthroplasty; mHKA—mechanical hip-knee-ankle-angle (angle between femoral and tibial mechanical axis); aHKA—arithmetic hip-knee-ankle-angle; LDFA—lateral distal femoral angle; MPTA—medial proximal tibial angle. [The figure is the author’s original work, arranged using vector graphic software (CorelDRAW Graphics Suite 21, Cascade Parent Limited, Ottawa, ON, Canada)].

**Figure 2:**
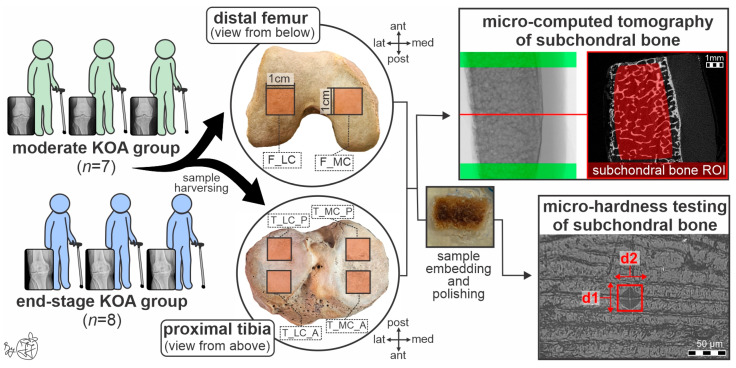
Study design and methodology used in the present study. Abbreviations: KOA—knee osteoarthritis; F_LC—lateral femoral condyle; F_MC—medial femoral condyle; T_LC_A—anterior part of lateral tibial condyle; T_LC_P—posterior part of lateral tibial condyle; T_MC_A—anterior part of medial tibial condyle; T_MC_P—posterior part of medial tibial condyle; ant—anterior; post—posterior; lat—lateral; med—medial; ROI—region of interest; d1,d2—indentation diagonals. [The figure is the author’s original work, created manually with vector graphic software (CorelDRAW Graphics Suite 21, Cascade Parent Limited, Ottawa, ON, Canada)].

To avoid the covariant effects of knee malalignment on the subchondral bone [12] and considering previous reports of less frequent valgus knee malalignment in Caucasian individuals with primary KOA [26,27,28], our study focused on individuals with varus knee malalignment, as confirmed by negative arithmetic hip-knee-ankle-angle values on preoperative long-standing radiographs (Figure 1A). Kellgren–Lawrence grading system using preoperative knee radiographs was employed to distinguish the severity of KOA lesions in the included individuals [29]. Kellgren–Lawrence stage 3 KOA was designated to individuals with moderate KOA (Figure 1B), characterized by multiple moderate osteophytes, definite moderate joint space narrowing, and subchondral sclerosis, while Kellgren–Lawrence stage 4 KOA was designated to individuals with end-stage KOA (Figure 1C), characterized by large osteophytes, severe to complete joint space narrowing, severe subchondral sclerosis, and visibly altered shape of the femur and tibia [29]. Thus, based on primary KOA severity, bone samples from included individuals (6 samples per person, Figure 2) were divided into the following two groups: moderate KOA group (7 patients, 42 obtained bone samples) and end-stage KOA group (8 patients, 48 obtained bone samples).

Cube-like bone samples (approximate size: 1 cm × 1 cm × 1 cm, Figure 2) were obtained using a slow-rotating cordless autopsy saw (Kugel Medical, Regensburg, Germany), manually cleaned of adherent soft tissue, submerged in 70% ethanol, residually cleaned using Sonocool 255 ultrasonic bath (Bandelin, Berlin, Germany), completely air-dried, dehydrated using ascending concentrations of ethanol solutions, resin-embedded (EpoThinTM slow-setting resin, Buehler, Braunschweig, Germany), and paper-polished (600, 1200, and 4000 grits) using a water-cooled polishing machine (EQ-Unipol 810 polishing machine; MTI Corporation, Richmond, CA, USA) before mechano-structural analysis of subchondral trabecular bone (Figure 2).

### 2.2. Micro-Computed Tomography Assessment

In the Center of Bone Biology, subchondral trabecular bone microarchitecture was evaluated using the Skyscan 1172 micro-computed tomography system (Bruker micro-CT, Skyscan, Kontich, Belgium). Bone samples were scanned and reconstructed according to our previous protocol recommendations to ensure adequate image quality and reliable standards for human bone micro-CT imaging [12,30]. Specifically, a spatial resolution of 10 μm, 80 kV, 126 μA, an Al + Cu filter, a 1332 × 2000-pixel camera, a 1200 ms exposure time, a 0.4° rotation step, and triple-frame averaging were employed for scanning. Appropriate corrections for misalignment, thermal drift, ring artifacts, and beam hardening were applied using NRecon software (version 1.7.4.6, Skyscan, Kontich, Belgium) to reconstruct bone volume.

To ensure comparability with previous studies [12], a total of 701 slices from each sample were included within the volume of interest in the subchondral trabecular bone, resulting in approximately 0.7 cm^3^ of tissue volume analyzed per sample. The volume of interest comprised auto-interpolated, manually demarcated regions of interest (ROI) in the subchondral bone of each sample (shown in red, Figure 2). A manual demarcation procedure was performed by a single author with years of experience in micro-CT assessment to minimize interobserver variability. The transitory cortico-trabecular zone and any marginal tissue damaged during cutting were consistently excluded during the manual ROI demarcation process (Figure 2). The following microarchitectural parameters were analyzed using the latest 64-bit CT-Analyzer software (CT.An 2020; version 1.20.30.0, Skyscan, Kontich, Belgium): bone volume fraction (BV/TV, %), trabecular thickness (Tb.Th, µm), trabecular separation (Tb.Sp, mm), trabecular number (Tb.N, 1/mm), structure model index (SMI, dimensionless), and connectivity density (Conn.Dn, 1/mm^3^). To evaluate the level of subchondral bone tissue mineralization, mean grayscale index (MGSI, dimensionless) analysis was performed using a previously published protocol for human bone [31]. Since reconstructed micro-CT images contain voxels with varying grayscale intensities that indicate different hydroxyapatite content, we used grayscale levels between 95 and 255 for MGSI analysis of subchondral bone.

### 2.3. Vickers Micro-Hardness Testing

In the Center of Bone Biology, the mechanical properties of the subchondral bone were evaluated using the Vickers micro-indentation testing device (HMV-G system, Shimadzu, Kyoto, Japan), following our previously established testing protocol for human bone [32]. Bone micro-hardness (the ability of bone to resist plastic deformation due to constant force applied during micro-indentation) was measured by Vickers micro-hardness (VmH, kg/mm^2^) with a load of 5 gf, a test duration of 12 s, a Vickers diamond indenter (that has a pyramidal tip with a 136° angle), and ×40 magnification [31,32]. The Vickers micro-hardness was automatically calculated using the formula VmH = 0.1891 × (F/d^2^), where the constant load force (F) and the average length of the indentation diagonals (d_1_ and d_2_, marked in Figure 2) were used. To reduce subjective bias, two researchers independently performed micro-indentation testing, and their average values were used in statistical analysis. To account for bone tissue heterogeneity, micro-hardness testing consisted of five effective indentations on the intact surfaces of the trabecular subchondral bone (Figure 2). Testing was performed with at least one field of view between the indentation sites to prevent overlapping effects and at least 60 µm away from the Haversian canal to avoid boundary effects. Indentation sites were randomly selected, ensuring that at least 1 mm^2^ of trabecular subchondral bone tissue was evaluated per sample. Tests were considered invalid if the horizontal and vertical indentation diagonals differed by more than 10% from the longer diagonal.

### 2.4. Statistical Analysis

The Kolmogorov–Smirnov test was used to assess the normality of the distribution in mechano-structural parameters. Levine’s test was used to assess the homogeneity of the data variance. Analysis of variance (ANOVA) for repeated measures was used to evaluate potential differences between the investigated stages of KOA (end-stage KOA group vs. moderate KOA group), between investigated skeletal sites (F_LC, F_MC, T_MC_A, T_MC_P, T_LC_A, and T_LC_P), and their interaction (groups were set as a between-subject factor, while skeletal sites were set as a within-subject factor). The percentage of difference between the KOA stage 4 group and the KOA stage 3 group in the analyzed regions was calculated to evaluate the stage-specific alterations in subchondral trabecular bone mechano-structure. Statistical analysis was performed using IBM SPSS Statistics (version 27.0; International Business Machines Corporation, Armonk, NY, USA) at a 0.05 significance level and 95% confidence interval.

## 3. Results

It is important to note that the moderate KOA group and the end-stage KOA group did not differ in age, height, weight, or body mass index (*p* > 0.05, Table 1). The total pool of individuals in the study was intended to include a similar proportion of conjoined conditions and diseases to prevent significant covariate effects on our results (Table 1).

**Figure 3:**
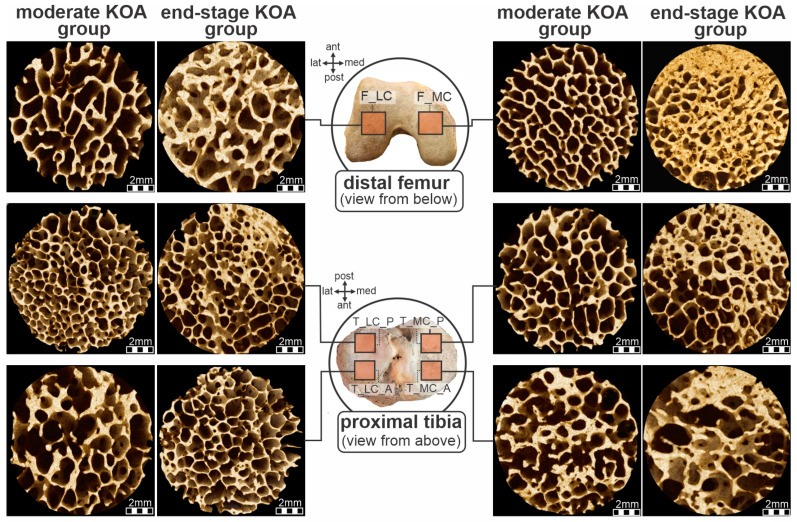
Representative findings of subchondral bone microarchitecture in the distal femur and proximal tibia of patients undergoing TKA due to moderate and end-stage KOA. Abbreviations: KOA—knee osteoarthritis; F_LC—lateral femoral condyle; F_MC—medial femoral condyle; T_LC_A—anterior part of lateral tibial condyle; T_LC_P—posterior part of lateral tibial condyle; T_MC_A—anterior part of medial tibial condyle; T_MC_P—posterior part of medial tibial condyle; ant—anterior; post—posterior; lat—lateral; med—medial. [The figure is the author’s original work, created with 3D rendering software (CT vox 3.1.2.0, Bruker, Belgium) and arranged with vector graphic software (CorelDRAW Graphics Suite 21, Cascade Parent Limited, Ottawa, ON, Canada)].

Our data revealed that the subchondral trabecular bone microarchitecture in the distal femur and proximal tibia differed significantly between the end-stage KOA and moderate KOA groups (Figure 3). Namely, our data revealed significantly higher trabecular BV/TV in bone samples collected from individuals with end-stage KOA compared to moderate KOA (group *p* = 0.040, Table 2), due to significantly thicker trabeculae (group *p* = 0.014) that were less separated (group *p* = 0.012) and better connected (higher Conn.Dn values, group *p* = 0.046, Table 2). Furthermore, higher values of MGSI and VmH values were noted in distal femur and proximal tibia samples harvested from individuals with KOA stage 4 compared to bone samples of the KOA stage 3 group (group *p* = 0.048 and group *p* = 0.015, respectively, Table 2). Despite a tendency towards increased Tb.N in the end-stage KOA group, these values were not significantly different compared to bone samples obtained from the moderate KOA group (group *p* > 0.05, Table 2). Furthermore, SMI analyses revealed a predominance of plate-like trabeculae in the subchondral bone of the obtained bone samples, without reaching statistical significance across KOA stages (group *p* > 0.05).

Furthermore, our data pointed towards site specificity in the subchondral trabecular bone microstructure, revealing the tendency towards high BV/TV (site *p* = 0.079), coupled with the high Tb.Th (site *p* = 0.040) and high MGSI (site *p* = 0.008) in bone samples obtained from the medial femoral and tibial condyles (site *p* < 0.05, Table 2). These data reveal a compensatory response to prominent mechanical load in the medial femoral and tibial condyles, associated with varus knee malalignment in individuals included in our study.

**Figure 4:**
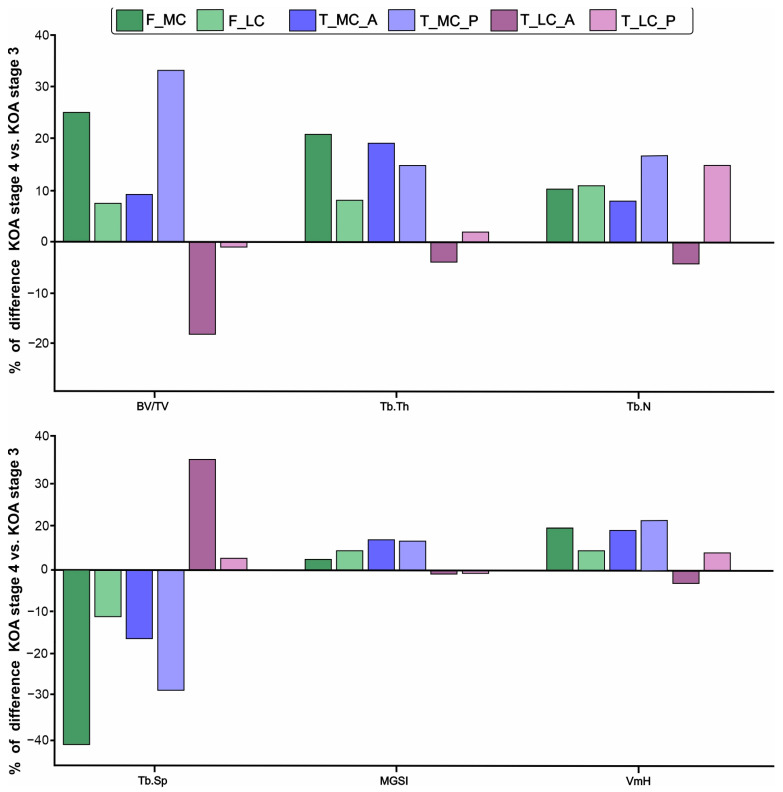
Stage-specific percentage of difference in subchondral bone microarchitectural parameters of distal tibia and proximal femora samples from individuals with primary KOA undergoing TKA. Abbreviations: KOA—knee osteoarthritis; F_LC—lateral femoral condyle; F_MC—medial femoral condyle; T_LC_A—anterior part of lateral tibial condyle; T_LC_P—posterior part of lateral tibial condyle; T_MC_A—anterior part of medial tibial condyle; T_MC_P—posterior part of medial tibial condyle; BV/TV—bone volume fraction; Tb.Th—trabecular thickness; Tb.Sp—trabecular separation; MGSI—mean grayscale index; VmH—Vickers micro-hardness. [The figure is the author’s original work, created with data analysis and graphing software (Origin 2018, OriginLab Corporation, Northampton, MA, USA)].

Our data also revealed that the KOA stage may induce site-specific effects on the mechano-structure of subchondral trabecular bone (Figure 4, Table 2). Namely, a tendency towards significant interaction was noted for Tb.Th (site × group *p* = 0.059), while significant interaction was noted for VmH values (site × group *p* = 0.02, Table 2), revealing that mechano-structural alterations were the most prominent in the medial femoral and tibial condyles of the end-stage KOA group. Namely, the most pronounced difference between individuals with KOA stage 4 and KOA stage 3 was observed for BV/TV, Tb.Th and Tb.Sp of samples obtained from medial tibial and femoral condyles (Figure 4). Further, our data indicated that the most heterogeneous mechano-structure was noted in the samples obtained from the medial tibial and femoral condyles (Figure 4). Despite a general trend toward sclerosis in the subchondral trabecular bone of individuals with end-stage KOA (Figure 3), our data showed a tendency toward lower BV/TV, Tb.Th, Conn.Dn and VmH, coupled with higher Tb.Sp in the anterior part of the lateral tibial condyle sampled from individuals with KOA stage 4 (Table 2).

## 4. Discussion

The subchondral trabecular bone is a key structural element of the knee joint and plays a significant role in the KOA progression [15]. Early subchondral trabecular bone remodeling, including changes in microarchitecture and metabolic activity, may precede cartilage degeneration, reinforcing the view of primary osteoarthritis as a whole-joint disease in which subchondral bone plays a central pathogenic role [15]. Our data demonstrate that progression from moderate to end-stage KOA (Kellgren–Lawrence stage 3 to Kellgren–Lawrence stage 4) is associated with pronounced alterations in subchondral trabecular bone microarchitecture, accompanied by changes in its material and mechanical properties (Table 2). Overall, end-stage KOA was characterized by higher trabecular BV/TV and Tb.Th, along with reduced Tb.Sp, suggesting that subchondral bone remodeling in end-stage KOA is characterized predominantly by thickening and coalescence of pre-existing trabeculae rather than by the formation of new trabecular elements, as recently reported [1,33,34]. In parallel with these microarchitectural changes, we also identified significantly higher MGSI and VmH values, indicating increased mineralization and mechanical stiffness of the subchondral bone in end-stage KOA compared to moderate KOA. Observed increase in MGSI and VmH supports the concept that radiographic density-related parameters reflect underlying micro-scale changes in bone properties [35]. Importantly, our results suggest a close relationship between bone mineral content, microarchitectural adaptation, and mechanical competence. These findings reflect that the subchondral bone sclerotic phenotype has played a pivotal role in the underlying pathogenesis of the disease in our individuals, as previously reported [36,37]. Furthermore, our micro-CT analysis revealed a predominance of plate-like trabecular appearance in the subchondral bone of patients with KOA, regardless of Kellgren–Lawrence stage, indicating their less flexible nature that can bear higher compressive loads with less deformation [38]. These data, combined with higher MGSI and VmH values indicating more brittle subchondral bone (high stiffness with low fracture toughness), suggest that local bone mechano-structural alterations in individuals with KOA may cause microdamage accumulation [33] and subsequently hamper implant stability, affecting postoperative outcomes in patients undergoing TKA [25]. Still, it is important to note that previous clinical studies recognized varus malalignment, ligament imbalance, chronic medial compartment overload, and tibial component malposition as dominant drivers of bone–cement interface failure, due to high shear and tensile stresses leading to aseptic loosening, debonding, and osteolysis [39,40,41]. These data indicate that the observed subchondral sclerosis may function more as a secondary amplifier, warranting further research to quantify the contribution of subchondral bone structure alterations to cement-bone interface quality in TKA.

Our data also indicated site-specific variation in subchondral bone mechano-structural properties, with the most pronounced variation in the medial femoral and tibial condyles of individuals with end-stage KOA. Our findings indicate increased local bone adaptation in regions exposed to greater mechanical stress, present in the typical varus malalignment of patients with KOA undergoing TKA [28]. It is important to note that a limited number of studies have addressed the femoral bone samples, and consequently, the available data on femoral mechano-structural features across KOA stages remain scarce. Our study demonstrated comparable, and in some instances even more pronounced, changes in the femoral samples, revealing one of the key strengths in highlighting the importance of analyzing not only the tibial but also the femoral articular surface. Similar findings were reported by Azari et al. [35], who demonstrated comparable microarchitectural characteristics of the femur and tibia in advanced KOA stages. In contrast, Beuf et al. [42] reported significant differences between femoral and tibial samples observed in the early stages of the disease. Furthermore, Hu et al. [21] recently showed compromised femoral mechanical properties of subchondral bone, with impaired bone quality, as the disease progresses, underscoring the need for further research to fully unravel this topic.

Our study is constrained by the inherent limitations of its cross-sectional design. The chosen study design involved a homogeneous group of patients with KOA undergoing TKA, indicating that the effects of various factors (smoking duration, presence of undiagnosed comorbidities, effects of chronic therapy, malnutrition, vitamin D levels, and valgus malalignment) that may be present in real-life clinical settings could not be adequately evaluated. Since we wanted to exclude load-induced covariant effects by including a homogeneous group of individuals with varus knee malalignment and without ligament instability, future studies should include individuals with neutral alignment of primary KOA to elucidate whether subchondral bone changes are more attributable to mechanical loads during knee malalignment or to KOA-stage-related changes. This study used negative arithmetic hip-knee-ankle-angle values on preoperative long-standing radiographs to confirm varus knee malalignment in the included individuals, while future studies are needed to evaluate the relationship between micro-scale subchondral bone alterations and the severity of knee malalignment in primary KOA. Further, our study relied on the Kellgren-Lawrence score to provide a static assessment of disease severity. Still, it would be beneficial to include the long-term implant survival analysis and longitudinal radiographic evaluation of potential loosening and bone-implant interference, which would provide additional insight into the clinical relevance of the observed subchondral bone changes. Future studies should consider integrating the association between histopathological scores of KOA severity and structural changes in subchondral bone [43]. Introducing contrast-enhanced micro-CT to evaluate the subchondral bone microvasculature would benefit elucidating its contribution to the observed KOA-stage-induced skeletal alterations [44,45]. Our study is also limited by the lack of assessment of bone remodeling, which precludes information about the relative contributions of increased bone resorption and decreased bone formation to the subchondral alterations observed in our individuals. Moreover, we performed an indirect assessment of subchondral bone mineral content using MGSI analysis because our facility lacks phantom calibration, necessitating further studies to fully explore this topic.

## 5. Conclusions

Our data revealed site-specific alterations in the mechano-structure of subchondral trabecular bone in individuals with end-stage KOA, indicating that substantial subchondral sclerosis is present in these individuals and supporting its role in primary KOA pathogenesis beyond its exclusive effect on cartilage degeneration. Namely, our data revealed thicker trabeculae, higher mineral content, and increased bone micro-hardness in tibial and femoral samples obtained from patients with end-stage KOA compared with those with moderate KOA, indicating its compensatory nature. Observed subchondral bone mechano-structural alterations could contribute to affected long-term implant stability and potentially harm treatment outcomes in individuals with primary KOA requiring TKA. Our data also revealed that the most heterogeneous mechano-structure was present in bone samples obtained from the medial tibial and femoral condyles, indicating a substantial effect of mechanical loading due to varus malalignment present in our patients.

## Figures and Tables

**Table 1:** Basic anthropometric and clinical data of the included individuals.

	Moderate KOA Group (*n* = 7)	End-Stage KOA Group (*n* = 8)
Basic anthropometric details of included patients		
Age (mean ± SD)	70 ± 7 years	70 ± 6 years
Height (mean ± SD)	169 ± 5 cm	168 ± 8 cm
Weight (mean ± SD)	89 ± 12 kg	85 ± 14 kg
Body Mass Index (mean ± SD)	31.2 ± 3.3 kg/m^2^	30.0 ± 3.0 kg/m^2^
Sex of the patient (*n*/max)	Females: 3/7 Males: 4/7	Females: 5/8 Males: 3/8
Knee malignment		
Varus knee malalignment (*n*/max)	7/7	8/8
Details about chronic comorbidities		
Overweight	3/7	4/8
Obesity class 1	4/7	4/8
Hypertension (*n*/max)	7/7	8/8
Coronary artery disease (*n*/max)	1/7	2/8
Peripheral vascular disease (*n*/max)	3/7	0/8
Hyperlipidemia (*n*/max)	1/7	2/8
Diabetes mellitus type 2 (*n*/max)	5/7	1/8
Thyroid disorders (*n*/max)	3/7	0/8

Abbreviations: KOA—knee osteoarthritis, SD—standard deviation.

**Table 2:** Comparative analysis of subchondral bone mechano-structural properties in individuals with moderate and end-stage KOA.

Skeletal Site		Moderate KOA Group (Mean ± SD)	End-Stage KOA Group (Mean ± SD)	*p* Value
bone volume fraction (%)	F_MC	27.67 ± 8.98	37.42 ± 6.66	group *p* = 0.040 site *p* = 0.079 site × group *p* = 0.408
	F_LC	25.93 ± 5.58	28.01 ± 9.30	
	T_MC_A	22.45 ± 10.93	24.72 ± 12.68	
	T_MC_P	19.66 ± 11.83	29.76 ± 18.71	
	T_LC_A	23.91 ± 10.41	20.17 ± 5.09	
	T_LC_P	23.23 ± 10.99	23.12 ± 9.93	
trabecular thickness (µm)	F_MC	157 ± 50	197 ± 14	group *p* = 0.014 site *p* = 0.040 site × group *p* = 0.059
	F_LC	157 ± 18	170 ± 50	
	T_MC_A	162 ± 62	199 ± 79	
	T_MC_P	165 ± 85	193 ± 73	
	T_LC_A	173 ± 75	166 ± 58	
	T_LC_P	184 ± 30	187 ± 50	
trabecular number (1/mm)	F_MC	1.73 ± 0.53	1.93 ± 0.36	group *p* = 0.898 site *p* = 0.148 site × group *p* = 0.504
	F_LC	1.61 ± 0.39	1.81 ± 0.71	
	T_MC_A	1.58 ± 0.84	1.72 ± 0.65	
	T_MC_P	1.42 ± 0.41	1.70 ± 0.57	
	T_LC_A	1.51 ± 0.46	1.46 ± 0.47	
	T_LC_P	1.51 ± 0.53	1.77 ± 0.77	
trabecular separation (mm)	F_MC	0.39 ± 0.13	0.19 ± 0.03	group *p* = 0.012 site *p* = 0.497 site × group *p* = 0.165
	F_LC	0.36 ± 0.12	0.32 ± 0.12	
	T_MC_A	0.43 ± 0.14	0.36 ± 0.17	
	T_MC_P	0.48 ± 0.17	0.35 ± 0.19	
	T_LC_A	0.35 ± 0.15	0.44 ± 0.14	
	T_LC_P	0.37 ± 0.17	0.38 ± 0.16	
connectivity density (1/mm^3^)	F_MC	24.54 ± 17.02	38.81 ± 17.20	group *p* = 0.046 site *p* = 0.327 site × group *p* = 0.225
	F_LC	28.15 ± 14.12	31.92 ± 18.46	
	T_MC_A	25.74 ± 15.28	26.93 ± 11.46	
	T_MC_P	26.44 ± 9.78	41.15 ± 27.89	
	T_LC_A	31.56 ± 11.58	23.30 ± 13.17	
	T_LC_P	31.69 ± 15.84	35.49 ± 14.34	
bone mean grayscale index (dimensionless)	F_MC	149.25 ± 3.17	152.17 ± 2.89	group *p* = 0.048 site *p* = 0.008 site × group *p* = 0.409
	F_LC	138.69 ± 6.68	143.79 ± 12.82	
	T_MC_A	139.77 ± 2.68	148.27 ± 2.92	
	T_MC_P	135.99 ± 14.98	143.69 ± 5.43	
	T_LC_A	143.63 ± 10.89	142.75 ± 13.42	
	T_LC_P	149.67 ± 3.92	148.72 ± 4.67	
Vickers hardness (kg/mm^2^)	F_MC	61.65 ± 3.38	75.74 ± 7.00	group *p* = 0.015 site *p* = 0.152 site × group *p* = 0.02
	F_LC	67.20 ± 8.82	73.69 ± 6.62	
	T_MC_A	58.72 ± 18.39	71.53 ± 8.24	
	T_MC_P	57.07 ± 14.43	72.91 ± 10.82	
	T_LC_A	69.56 ± 4.51	65.96 ± 7.02	
	T_LC_P	67.54 ± 9.85	73.11 ± 6.61	

Abbreviations: KOA—knee osteoarthritis; F_LC—lateral femoral condyle; F_MC—medial femoral condyle; T_LC_A—anterior part of lateral tibial condyle; T_LC_P—posterior part of lateral tibial condyle; T_MC_A—anterior part of medial tibial condyle; T_MC_P—posterior part of medial tibial condyle.

## Data Availability

The original contributions presented in this study are included in the article. Further inquiries about data availability should be directed to the corresponding author (J.J.).
